# White fat, factitious hyperglycemia, and the role of FDG PET to enhance understanding of adipocyte metabolism

**DOI:** 10.1186/2191-219X-1-2

**Published:** 2011-06-07

**Authors:** Michael S Hofman, Rodney J Hicks

**Affiliations:** 1 Center for Cancer Imaging, Peter MacCallum Cancer Centre, St. Andrews Place, East Melbourne, VIC 3002 Australia; 2 Departments of Medicine and Radiology, University of Melbourne, Melbourne, VIC Australia

## Abstract

The development of a hybrid PET/CT led to the recognition of the enhanced glycolysis in brown fat. We report a previously unrecognized mechanism for altered fluorodeoxyglucose (FDG) biodistribution with diffuse white adipose tissue uptake. This occurred during a restaging scan for cervical cancer following administration of insulin in the setting of measured hyperglycemia. The patient's blood sugar normalized, but she experienced symptoms and signs of hypoglycemia. A subsequent history indicated that the patient received intravenous high-dose vitamin C just prior to arrival. Ascorbic acid is a strong reducing agent and can cause erroneous false positive portable glucometer readings. Accordingly, it is likely the patient was euglycemic on arrival and was administered FDG during a period of insulin-induced hypoglycemia. Prominent diffuse white adipose tissue, gastric mucosal, myocardial, and very low hepatic and muscle activity were observed. The case provides insight into the metabolic changes that occur during hypoglycemia and the potential danger of relying on portable glucometer readings. We discuss the potential biological basis of this finding and provide recommendations on the avoidance of this complication.

## Background

The development of hybrid positron emission tomography/computed tomography (PET/CT) devices led to the recognition of enhanced glycolysis in brown fat, typically in the neck and paravertebral regions of the thorax and upper abdomen, as a thermoregulatory response and under catecholamine stimulation [1-3]. Other atypical patterns of fat uptake in patients with lipodystrophy have been reported [2,3] We report a previously unrecognized mechanism for altered fluorodeoxyglucose (FDG) biodistribution into adipose tissues.

## Case presentation

A 40-year-old woman presented for restaging with F-18 FDG PET/CT on the background of squamous cell carcinoma of the cervix and biopsy proven recurrence in a left supraclavicular node. Conventional imaging had demonstrated no further evidence of metastatic disease. She had previously received radical chemoradiotherapy for FIGO stage IIIb disease with para-aortic nodal involvement, without intervening therapy in the 6 months since completing the treatment. The patient was not diabetic and had a body mass index of 24, which is in the normal range for a female. She had no other past medical history, took no medications, and had fasted from the previous evening.

On arrival, her blood glucose level (BGL) obtained with a portable glucometer (Abbott Diabetes Care Optium Xceed, Alameda, CA, USA) via a fingerprick capillary blood sample was 15 mmol/L, and she was administered 6 U of short-acting insulin intravenously as per local protocol. Thirty minutes later, her BGL had fallen to 6.9 mmol/L, and she described feeling unwell with anxiety, palpitations, and sweating. Her blood pressure, temperature, and oxygen saturation levels were normal, and she was observed. The BGL measurements plateaued at 6.0 mmol/L, and FDG was subsequently injected. PET/CT scanning was performed 60 min later.

**Figure 1:**
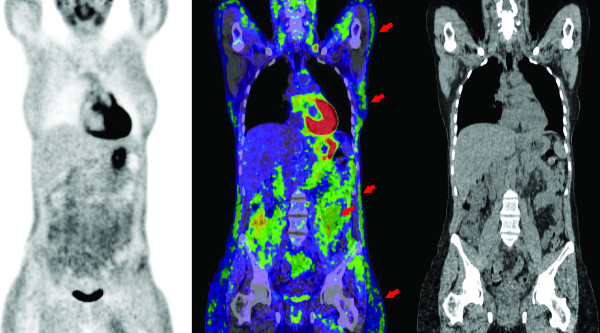
**Biodistribution of FDG**. Coronal PET, PET/CT fusion, and CT images demonstrating prominent subcutaneous white adipose tissue metabolic activity throughout the thorax, abdomen, and pelvis (arrows). More intense metabolic activity uptake was observed in intra-abdominal fat (arrow). Intense gastric and myocardial uptake combined with low hepatic and negligible muscle uptake was observed.

The images demonstrated altered biodistribution of FDG with a prominent uptake of the radiotracer throughout white adipose tissue (WAT), gastric mucosa, myocardium and very low hepatic activity (standardized uptake value (SUVmax) 2.3, 5.0, 16, and 1.2, respectively) (Figure 1). WAT was most prominent in intra-abdominal (mesenteric) fat. There was negligible muscle uptake (SUVmax, 1.0).

Given this unusual biodistribution, we questioned the patient further. She reported receiving an intravenous infusion of high-dose vitamin C (sodium ascorbate solution) from another health practitioner just prior to her arrival for the PET scan. Ascorbic acid is a strong reducing agent and interferes with laboratory tests involving oxidation and reduction reactions. Substantially reduced or elevated portable glucometer readings occur with ascorbic acid in a dose-dependent fashion and is one of the most common interfering substances that affects the accuracy of glucose meters [4,5]. A plasma venous sample was not available to confirm either plasma glucose or ascorbic acid as blood was obtained via fingerprick, and the error was not suspected prospectively. Nevertheless, the clinical symptoms of hypoglycemia and a history of intravenous ascorbic acid just prior to arrival at the PET scan, provides sufficient evidence to indicate that the patient was injected with FDG during a period of iatrogenic hypoglycemia induced by administration of insulin in the setting of a falsely elevated BGL reading.

**Figure 2:**
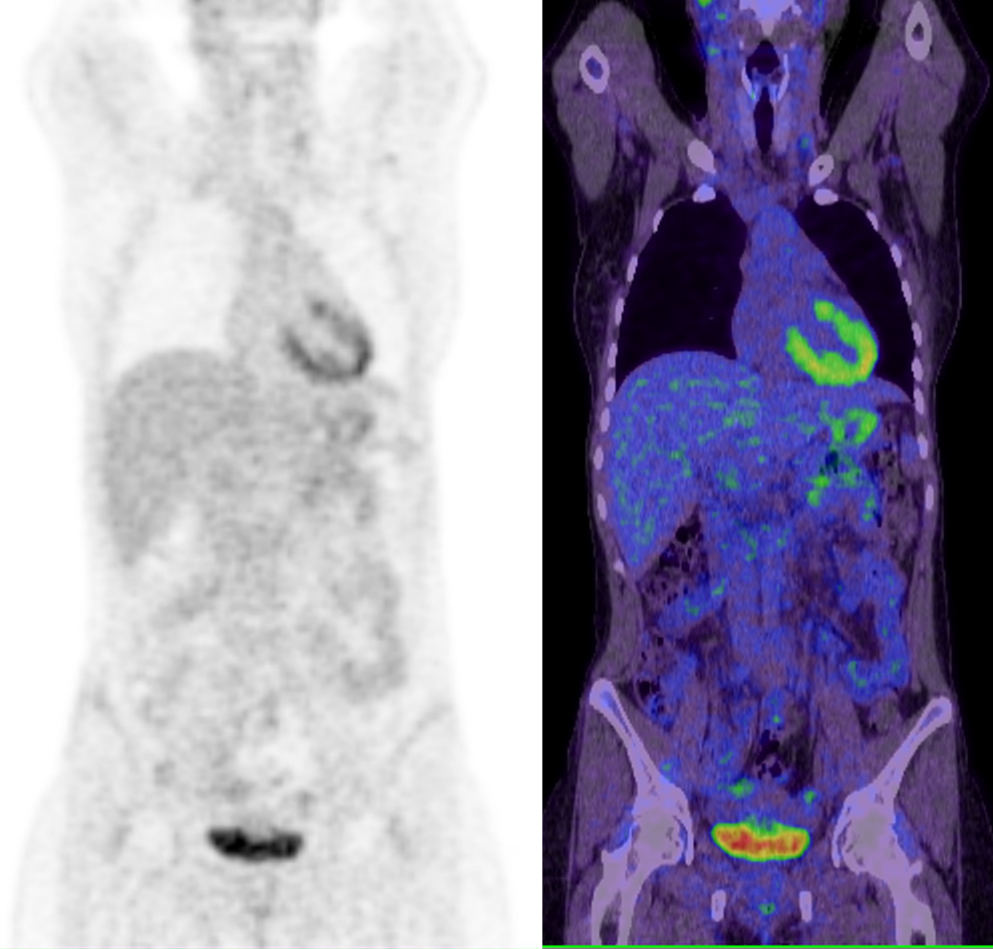
**Results of repeated study on biodistribution of FDG**. Coronal PET and PET/CT fusion images demonstrating normal distribution of FDG in the study performed the following day when normoglycemic in the absence of prior ascorbic acid or exogenous insulin.

Based on the unusual pattern of uptake, the study was repeated the following day in the absence of vitamin C. BGL was normal on arrival, and the FDG biodistribution was normal on the repeat study (see Figure 2; Additional file 1).

## Discussion

This case highlights the potential limitations of standardized insulin protocols [6,7], especially when relying on point-of-care glucometers. Intravenous ascorbic acid may result in substantial error in glucometer readings [4,5]. This is of particular relevance for cancer imaging as some complementary care practitioners advocate the use of vitamin C in conjunction with chemotherapy or radiotherapy in a wide variety of malignancies [8]; moreover, many patients choose not to tell their doctors about concomitant use of alternative medicines [9]. Clinicians should also be aware of other potential causes of error resulting in factitious hyperglycemia including substances containing maltose (e.g., intravenous human immunoglobulin) [10], icodextrin (e.g., peritoneal dialysis solution) [11] and galactose. Indeed, several deaths have been reported, and warnings have been issued by health regulatory agencies [12-15]. Accordingly, a patient should be questioned regarding the use of not only conventional chemotherapy and mediations but also whether they are having alternative therapies.

The case also illustrates a remarkable pattern of prominent WAT glycolytic activity on FDG PET. We hypothesize that this was likely physiologic and in response to hypoglycemia induced by administration of insulin. This is different than the characteristic pattern of diffuse muscular uptake visualized in hyperinsulinemic patients, during hyperinsulinemic euglycemic clamping or following oral glucose loading for optimization of cardiac imaging [16], or in nonfasted or hyperglycemic patients undergoing oncologic imaging [6]. It is also different than the pattern of FDG uptake observed in brown fat adipose tissue in cervical, supraclavicular, paravertebral regions, mediastinal, and suprarenal regions [1-3].

During hypoglycemia, major changes in metabolism occur, including mobilization of liver glycogen and release of energy stored in WAT into circulation as nonesterified fatty acids. Our findings demonstrate relative high-glucose uptake into adipocytes in response to hypoglycemia in a distribution consistent with WAT activation. The two main defenses to hypoglycemia are an increase in glucagon secretion and adrenaline secretion [17]. Glucagon, secreted by pancreatic α-cells, results in the stimulation of adenylate cyclase activity within adipocytes and plays the primary role in counter hormone regulation by promoting lipolysis in WAT [18-20]. Lipolysis also occurs via adrenaline released from the sympathetic nervous system terminals innervating WAT [21]. Catecholamines also activate brown fat with beta-blockers having been advocated as an intervention to reduce this confounding finding on FDG PET in predisposed individuals [22]. Other growth hormones such as FGF-21 may also play role. FGF-21 has been shown to stimulate glucose uptake in adipocytes and suppress hepatic glucose production [23]. Elevated FGF-21 levels have also been described in patients with HIV-associated lipodystrophy [24], where atypical FDG fat distribution is also described [25,26].

An additional hypothesis is that high-dose ascorbic acid administered in a short time period prior to the study is responsible, possibly in part, for the observed increased WAT glycolytic activity. Ascorbic acid dietary supplementation has been shown to reduce abdominal and subcutaneous fat depots in high-fat diet-induced adiposity animal models [27]. This study demonstrated upregulation of genes involved in cell proliferation and downregulation of genes participating in lipid metabolism and steroidogenesis in rats supplemented with ascorbic acid.

At the sites of increased WAT metabolic activity, a higher Hounsfield unit (HU) on CT was observed in the same region of WAT compared to the study performed one day later. Although not visually discernable, HU averaged -71 within WAT compared to -86 on the second scan, with an identical HU in other tissues such as muscle. This phenomenon has been described in brown adipose tissue (BAT) but, to the best of our knowledge, has not been described before in WAT. A prior animal and patient study demonstrated that the total lipid content of BAT was substantially decreased when activated under cold conditions, with a corresponding increase in CT HUs [28]. In this case, a rapid consumption of stored lipid within WAT may also account for the change seen. Greater blood flow in activated fat may also contribute to the rise in HU [29].

## Conclusion

FDG PET/CT is a useful noninvasive imaging modality for visualizing metabolic changes within adipocytes. A greater understanding of the role of both brown and white adipocyte tissue as endocrine organs is of public health interest as they may be central to our improved understanding of obesity and diabetes mellitus [30]. The mechanisms of observed WAT glycolytic activity in this case study are proposed but uncertain. In particular, the role of exogenous insulin, physiologic hormonal responses to hypoglycemia or ascorbic acid in inducing WAT glycolytic activity is uncertain. Further controlled studies utilizing FDG and tracers that interrogate other metabolic and receptor pathways may enhance our understanding of endocrine pathophysiology.

## Consent

Written informed consent was obtained from the patient for the publication of this manuscript and accompanying images. A copy of the written consent is available for review by the Editor-in-Chief of this journal.

## Competing interests

The authors declare that they have no competing interests.

## Authors' contributions

MSH and RJH both made contributions to conception and design, drafting, and revising the manuscript. All authors read and approved the final manuscript.

## Supplementary Material

Additional file 1 **Comparative rotating maximum intensity projection images demonstrating altered FDG biodistribution on the first study (right), with normalization on FDG biodistribution when repeated the following day in the absence of prior ascorbic acid or exogenous insulin (left)**. Click here for file
